# Identification of an *Acinetobacter baumannii* Zinc Acquisition System that Facilitates Resistance to Calprotectin-mediated Zinc Sequestration

**DOI:** 10.1371/journal.ppat.1003068

**Published:** 2012-12-06

**Authors:** M. Indriati Hood, Brittany L. Mortensen, Jessica L. Moore, Yaofang Zhang, Thomas E. Kehl-Fie, Norie Sugitani, Walter J. Chazin, Richard M. Caprioli, Eric P. Skaar

**Affiliations:** 1 Department of Pathology, Microbiology and Immunology, Vanderbilt University, Nashville, Tennessee, United States of America; 2 Mass Spectrometry Research Center, Vanderbilt University, Nashville, Tennessee, United States of America; 3 Department of Chemistry, Vanderbilt University, Nashville, Tennessee, United States of America; 4 Center for Structural Biology, Vanderbilt University, Nashville, Tennessee, United States of America; 5 Department of Biochemistry, Vanderbilt University, Nashville, Tennessee, United States of America; Tufts University School of Medicine, United States of America

## Abstract

*Acinetobacter baumannii* is an important nosocomial pathogen that accounts for up to 20 percent of infections in intensive care units worldwide. Furthermore, *A. baumannii* strains have emerged that are resistant to all available antimicrobials. These facts highlight the dire need for new therapeutic strategies to combat this growing public health threat. Given the critical role for transition metals at the pathogen-host interface, interrogating the role for these metals in *A. baumannii* physiology and pathogenesis could elucidate novel therapeutic strategies. Toward this end, the role for calprotectin- (CP)-mediated chelation of manganese (Mn) and zinc (Zn) in defense against *A. baumannii* was investigated. These experiments revealed that CP inhibits *A. baumannii* growth *in vitro* through chelation of Mn and Zn. Consistent with these *in vitro* data, Imaging Mass Spectrometry revealed that CP accompanies neutrophil recruitment to the lung and accumulates at foci of infection in a murine model of *A. baumannii* pneumonia. CP contributes to host survival and control of bacterial replication in the lung and limits dissemination to secondary sites. Using CP as a probe identified an *A. baumannii* Zn acquisition system that contributes to Zn uptake, enabling this organism to resist CP-mediated metal chelation, which enhances pathogenesis. Moreover, evidence is provided that Zn uptake across the outer membrane is an energy-dependent process in *A. baumannii*. Finally, it is shown that Zn limitation reverses carbapenem resistance in multidrug resistant *A. baumannii* underscoring the clinical relevance of these findings. Taken together, these data establish Zn acquisition systems as viable therapeutic targets to combat multidrug resistant *A. baumannii* infections.

## Author Summary

*Acinetobacter baumannii* is a bacterium responsible for an increasing number of infections in the hospital setting. These infections are particularly challenging because most strains of *A. baumannii* are resistant to commonly used antibiotics. Unfortunately, there is relatively little known about this organism and how it causes disease, making it difficult to identify new drug targets. In order to address this problem we examined the role for nutrient manganese (Mn) and zinc (Zn) in *A. baumannii* infections. We have determined that the host protein, calprotectin (CP), contributes to defense against *A. baumannii* pneumonia through chelation of nutrient Mn and Zn. Moreover, employing purified calprotectin as a probe allowed us to identify a Zn acquisition system in *A. baumannii* that is required for efficient Zn uptake *in vitro* and full pathogenesis *in vivo*. Finally, we demonstrate that inhibiting Zn acquisition can reverse antibiotic resistance mechanisms that rely on Zn-dependent enzymes. Taken together, these results demonstrate the importance of Zn acquisition to *A. baumannii* pathogenesis and antibiotic resistance, establishing Zn acquisition as a potential target for therapeutic development.

## Introduction

*Acinetobacter baumannii* is an opportunistic pathogen of growing importance in the hospital setting. Responsible for up to 20 percent of infections in intensive care units worldwide, *A. baumannii* is particularly problematic due to its propensity to acquire antibiotic resistance determinants [1]–[6]. Moreover, the resistance of *A. baumannii* to common disinfectants and ability to survive for long periods on dry surfaces make it difficult to eradicate from the hospital environment [7]–[10]. Current multidrug resistance rates range from 48–85% of isolates, with the greatest burden in Asia and Eastern Europe [1]–[5]. Pan resistance is likewise emerging, suggesting that more clinicians will soon be faced with infections for which no effective antimicrobial therapies remain [11]–[15]. Clearly, it is imperative to develop new antimicrobial strategies to combat this emerging threat.

Despite the growing clinical burden of *A. baumannii* disease there remains relatively little known about the mechanisms of *A. baumannii* pathogenesis or about this organism's physiologic requirements during infection [16]. However, it is established that all bacteria require certain transition metals in order to carry out basic physiologic functions [17]. Moreover, mammalian hosts take advantage of this requirement by limiting the availability of metals in a process referred to as nutritional immunity [17]–[19]. Although nutritional immunity was first used to describe the withholding of iron from invading bacteria, more recently it has been established that mammalian hosts also sequester manganese (Mn) and zinc (Zn) [17], [20]–[24]. As a result, bacterial pathogens need efficient mechanisms to acquire these metals from their hosts in order to cause disease. The contribution of host-mediated Mn and Zn limitation to defense against pulmonary infection has not been elucidated, and the bacterial processes that combat this host defense in the lung have not been described. In the case of *A. baumannii*, mechanisms for iron acquisition through siderophore biosynthesis and transport machinery have been identified [25]–[30]; however the mechanisms for acquiring other metals, such as Mn and Zn, remain to be uncovered.

Calprotectin (calgranulin A/B, MRP 8/14) (CP) is a member of the S100 class of EF-hand proteins consisting of a heterodimer of S100A8 and S100A9. CP is an important inflammatory marker and exhibits antimicrobial activity through the chelation of Mn and Zn [21], [31]–[33]. As a result, CP has been implicated in defense against a variety of bacterial and fungal pathogens [20]–[24]. CP comprises up to 50% of the neutrophil cytoplasmic protein content; therefore CP accumulates at sites of infection [34]. Neutrophils are a critical component of the innate response to *A. baumannii* infection suggesting that CP may play a role in defense against *A. baumannii in vivo* [35], [36]. Herein, we demonstrate that CP is abundantly expressed in the murine lung following infectious challenge and that this protein is involved in protection against *A. baumannii* pneumonia and dissemination to other organs. Using CP as a probe uncovered a number of genes required for resistance to Mn and Zn limitation and enabled the discovery of a Zn acquisition system in *A. baumannii.* This system is expressed in low Zn environments, required for Zn acquisition, and important for colonization of the lung. Finally, we show that an inability to acquire Zn reduces Zn-dependent antibiotic resistance mechanisms, increasing the sensitivity of *A. baumannii* to clinically relevant antimicrobials.

## Results

### CP contributes to defense against *A. baumannii* infection

**Figure 1:**
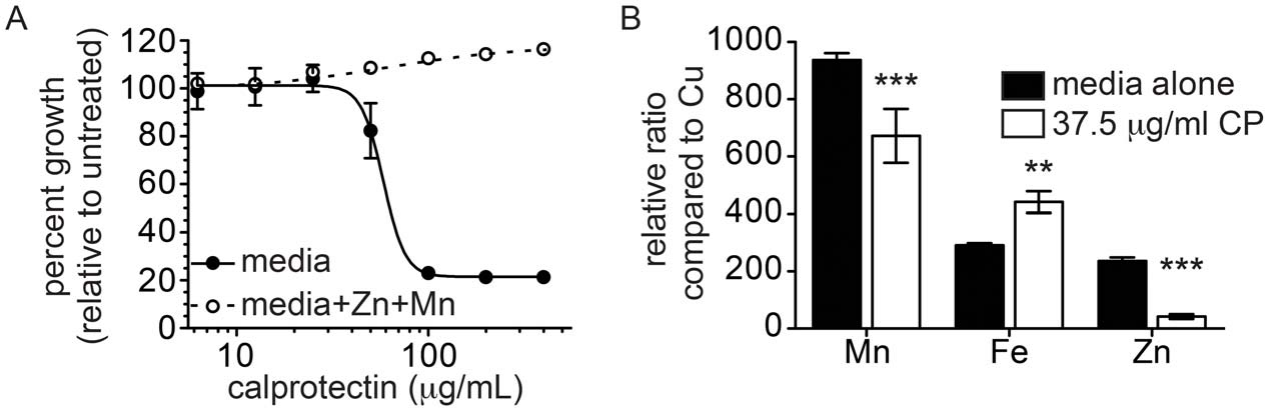
CP inhibits *A. baumannii* growth *in vitro* through chelation of Mn and Zn. (**A**) *A. baumannii* growth in the presence of increasing concentrations of CP with (dashed lines) or without (solid lines) 25 µM Mn and 25 µM Zn added back. Data represent the average of three biological replicates. (**B**) Treatment with CP reduces intracellular Mn and Zn accumulation. ICP-MS analyses of intracellular Mn, Fe and Zn expressed as the relative ratio compared to intracellular Cu. ** *p*<0.01, *** *p*<0.001 by two-way ANOVA.

As a first step toward evaluating the role for CP during *A. baumannii* infection, growth inhibition assays were performed with increasing concentrations of CP in growth media as described in the Materials and Methods (**Figure 1a**). In these conditions, CP inhibits *A. baumannii* growth with an IC_50_ of approximately 60 µg/ml. Importantly, the inhibitory effect of CP is completely reversed by the addition of excess Mn and Zn. Moreover, a variant of CP in which the Mn and Zn binding sites are mutated is unable to inhibit *A. baumannii* growth (**Figure S1**) [22]. Finally, CP exposure reduces intracellular accumulation of Mn and Zn, but not Fe, consistent with a role for CP in sequestering Mn and Zn away from *A. baumannii* (**Figure 1b**). Taken together, these results establish that CP inhibits *A. baumannii* growth through chelation of Mn and Zn.

**Figure 2:**
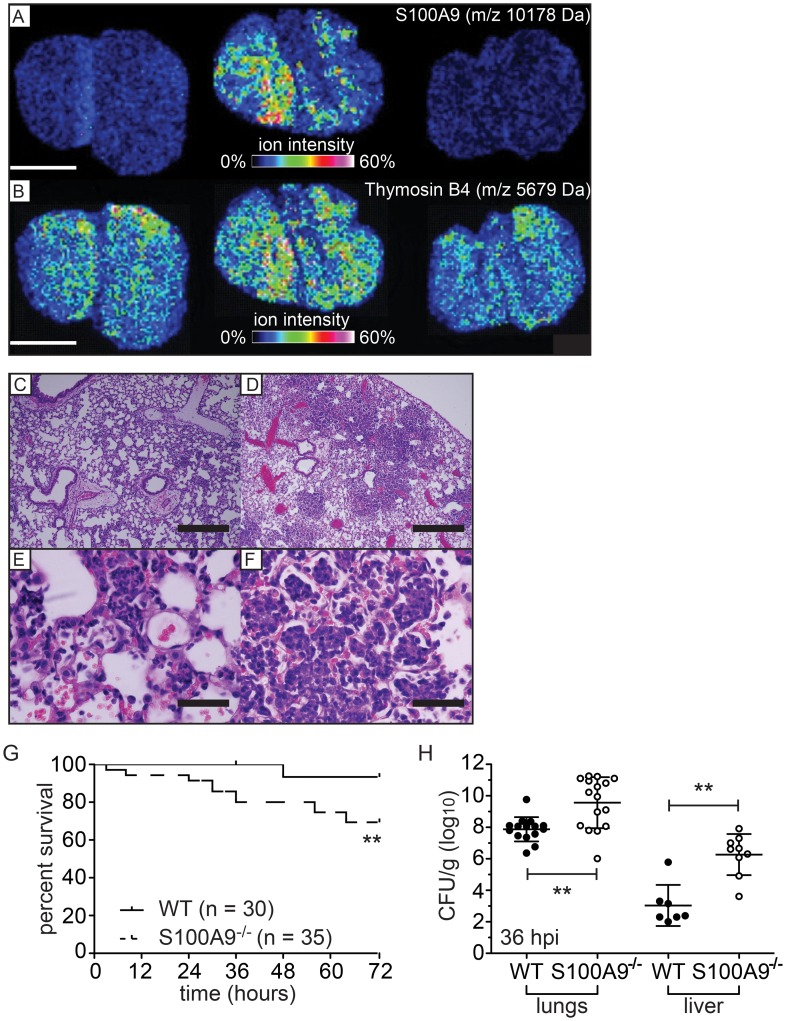
CP contributes to protection against *A. baumannii* infection. (**A–B**) IMS detection of host proteins in lungs harvested at 36 hpi with *A. baumannii.* Scale bar = 2 cm. (**A**) Heat map demonstrating the distribution and abundance of S100A8 (*m/z* = 10,165.8±5 Da) in uninfected wildtype (left) and infected wildtype (middle) or S100A9^−/−^ (right) mice. (**B**) Heat map demonstrating the distribution and abundance of a protein exhibiting *m/z* 5,679±5 Da in uninfected wildtype (left) and infected wildtype (middle) or S100A9^−/−^ (right) mice. (**C–F**) Hematoxylin and eosin stained histological sections taken from lungs harvested at 36 hpi from mice infected with *A. baumannii*. (**C, E**) Wildtype C57BL/6. (**D, F**) S100A9^−/−^. Scale bars equal 500 µm (C–D) and 50 µm (E–F). Images are representative of sections taken from three mice for each genotype. (**G**) Survival of wildtype or CP-deficient mice following infection with *A. baumannii*. Data were combined from three independent experiments with 8–16 mice per group in each experiment. ** *p*<0.01 as determined by Gehan-Breslow-Wilcoxon Test. (**H**) Bacterial burden in lungs and livers of wildtype or CP-deficient mice 36 hpi with *A. baumannii*. Data were averaged from three independent experiments with 5–10 mice per group in each experiment. ** *p*<0.01 as determined by Student's *t* test.

In order to define the contribution of CP to defense against *A. baumannii* pneumonia, we first determined whether CP is present in lungs of mice infected with *A. baumannii.* CP expression was measured using matrix-assisted laser desorption/ionization Imaging Mass Spectrometry (MALDI IMS). *In situ* visualization of CP by MALDI IMS has been previously characterized by our laboratory where CP is identified by the signal at *m/z* 10,165 corresponding to S100A8 [20], [37]. These experiments revealed that CP is not detectable by MALDI IMS in the lungs of uninfected wildtype C57BL/6 mice. In contrast, a robust signal for *m/z* 10,165 is observed in the lungs of wildtype mice infected with *A. baumannii*, demonstrating that CP is abundantly expressed in the lungs at 36 hours post infection (hpi) (**Figure 2a**). S100A9^−/−^ mice, which are functionally CP-deficient, do not exhibit a signal at *m/z* 10,165, establishing the specificity of these analyses (**Figure 2a**) [38]. To confirm that the failure to detect calprotectin in S100A9^−/−^ mice is not due to insufficient tissue or a generalized diminished protein signal in these sections we analyzed the signal at *m/z* 5,679 whose abundance is not affected by infection (**Figure 2b**) [39]. This signal did not differ between wildtype and S100A9^−/−^ mice demonstrating the biological relevance of the differential signal at *m/z* 10,165. Histological analyses of lung sections demonstrated robust inflammatory cell recruitment to the infected lungs in both wildtype and S100A9^−/−^ mice (**Figure 2c–f**). However, in S100A9^−/−^ lungs, the infectious and inflammatory foci involve more of the total lung area and more alveoli are packed with neutrophils as compared to wildtype mice. Taken together, these data demonstrate that CP accumulation in the lungs of wildtype mice coincides with neutrophil accumulation at sites of infection.

To determine the contribution of CP to protection against bacterial pneumonia, the susceptibility of S100A9^−/−^ mice to *A. baumannii* infection was compared to that of wildtype C57BL/6 mice. Although *A. baumannii* rarely causes lethal infection in immunocompetent mice, S100A9^−/−^ mice exhibit a significant increase in mortality over a 72-hour time course (**Figure 2g**). Consistent with this observation, bacterial burdens are significantly higher at 36 hpi in the lungs of S100A9^−/−^ mice compared to wildtype (**Figure 2h**). Moreover, dissemination to secondary sites is also increased in CP-deficient mice as evidenced by an increase in bacterial burden in livers. A significant increase in bacterial burden was not observed at 72 hpi in S100A9^−/−^ mice suggesting that mice that survive to 72 hours are eventually able to control infection (**Figure S2**). These data implicate CP as an important component of the innate immune response to *A. baumannii* pulmonary infections.

### Identification of *A. baumannii* mutants with altered sensitivity to CP

**Figure 3:**
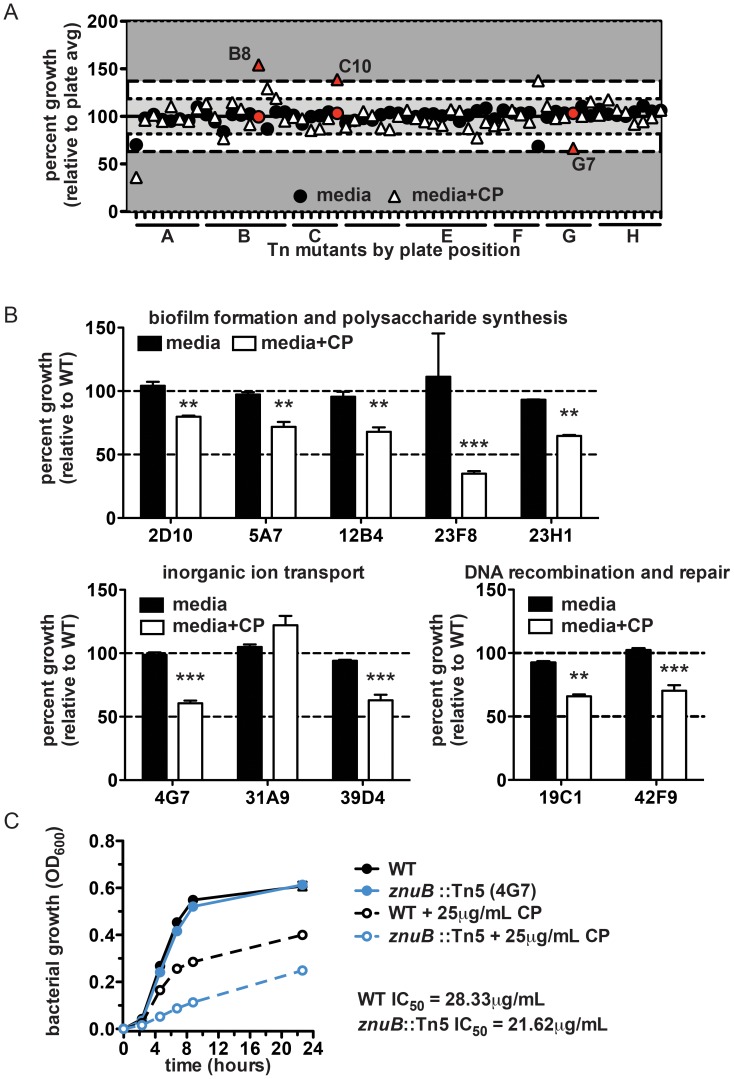
Transposon library screen to identify bacterial processes affected by CP treatment. (**A**) Bacterial growth from a representative plate of mutants cultured in media alone or media with 40 µg/ml CP. Data for wells in which no bacterial growth was observed in either condition have been omitted. Red symbols indicate mutants selected for secondary screening (see Materials and Methods). (**B**) Growth of selected mutants relative to wildtype bacteria cultured in media alone or media containing 40 µg/ml CP. Mutants are classified according to the predicted functional category of the gene disrupted by the transposon. ** *p*<0.01, *** *p*<0.001 for relative growth in the presence of CP compared to media alone, as determined by two-way ANOVA. (**C**) Growth curves comparing wildtype *A. baumannii* and *znuB*::Tn5 in the presence or absence of 25 µg/ml CP. IC_50_ values were determined as described in the Materials and Methods.

The contribution of CP to defense against *A. baumannii* infection suggests that *A. baumannii* requires Mn and/or Zn in order to maximally colonize the murine lung. Moreover, CP can serve as a valuable reagent to interrogate the contribution of Mn and Zn acquisition to *A. baumannii* physiology and pathogenesis. In order to determine the impact of Mn and Zn limitation on *A. baumannii* physiological processes, a transposon library was screened to identify mutants with either increased or decreased resistance to CP (**Figure 3a**). Approximately 4000 mutants were screened and 40 were selected whose growth was significantly different from wildtype in the presence of CP, but unchanged compared to wildtype in the absence of CP. Interestingly, a majority of the mutants cluster into a few common functional categories based on the annotations of the proteins encoded by the disrupted genes (**Figure 3b** **and** **Table 1**). These categories include biofilm formation and polysaccharide production, inorganic ion transport, and DNA replication or repair suggesting that these processes are involved in defense against CP.

**Table 1:** Transposon mutants with altered sensitivity to CP.

Mutant ID	Locus tag	Description	Phenotype	Insert type
PDL05G12	A1S_2148	flavin reductase-like protein; putative acetyl-CoA synthetase/AMP-(fatty) acid ligase	resistant	intergenic
PDL31A10	A1S_0352	hypothetical protein	resistant	intergenic
PDL02D10	A1S_2505; A1S_2506	hypothetical protein (110 bp 5′); putative GGDEF family protein (290 bp 3′)	sensitive	intergenic
PDL23H1	A1S_2841; A1S_2842	putative type 4 fimbrial biogenesis protein FimT (not present in the SEED); Acetyl-CoA C-acyltransferase	sensitive	intergenic
PDL15F1	A1S_3277	putative pirin-like protein	sensitive	intergenic
PDL03F9	A1S_0367	glutathione-regulated potassium-efflux system protein (K(+)/H(+) antiporter) (KefB)	resistant	intragenic
PDL03H9	A1S_0367	glutathione-regulated potassium-efflux system protein (K(+)/H(+) antiporter)	resistant	intragenic
PDL03H10	A1S_0367	glutathione-regulated potassium-efflux system protein (K(+)/H(+) antiporter)	resistant	intragenic
PDL05D12	A1S_0196	Long-chain-fatty-acid-CoA ligase	resistant	intragenic
PDL06C3	A1S_3463	Cro-like protein (pAb1)	resistant	intragenic
PDL26D12	A1S_2040	putative phage integrase	resistant	intragenic
PDL31A11	A1S_1053	hypothetical protein	resistant	intragenic
PDL31A9	A1S_0118	hypothetical protein	resistant	intragenic
PDL11C6	A1S_3142	putative membrane protein	resistant	intragenic
PDL04G7	A1S_0143	high affinity Zn transport protein	sensitive	intragenic
PDL06H4	A1S_2477	isocitrate dehydrogenase	sensitive	intragenic
PDL09F4	A1S_0076	aconitate hydratase	sensitive	intragenic
PDL05A7	A1S_0430	Putative glycosyltransferase	sensitive	intragenic
PDL19C1	A1S_2588	Holliday junction DNA helicase RuvB	sensitive	intragenic
PDL22A12	A1S_0023	putative malic acid transport protein	sensitive	intragenic
PDL23E2	A1S_3472	DNA replication protein (pAB2)	sensitive	intragenic
PDL23F8	A1S_0060	hypothetical protein	sensitive	intragenic
PDL12B4	A1S_0749	BfmS	sensitive	intragenic
PDL42C3	A1S_3352; A1S_3353	putative OHCU decarboxylase; putative transthyretin-like protein precursor	sensitive	intragenic
PDL42F9	A1S_0696	putative MutT/nudix family protein	sensitive	intragenic
PDL39D4	A1S_0118	hypothetical protein	sensitive	intragenic

### Identification of a Zn uptake system in *A. baumannii*

**Figure 4:**
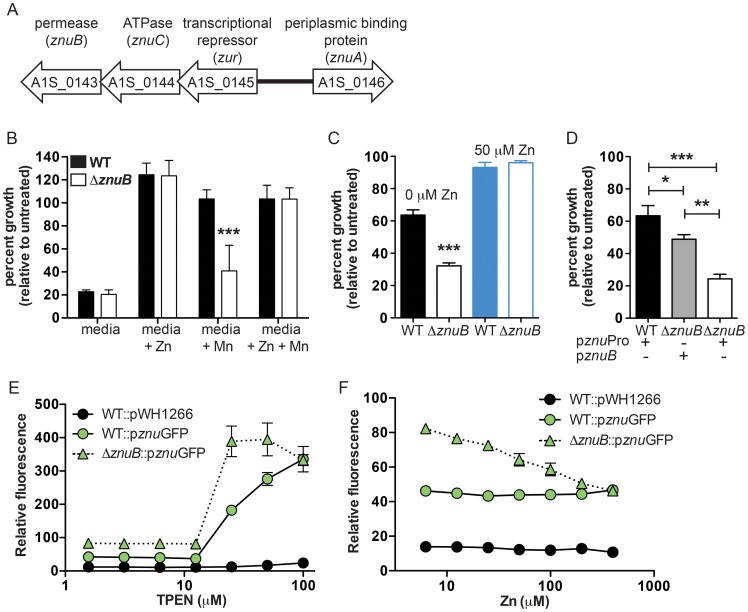
Contribution of *znuB* to growth in Zn-limiting conditions. (**A**) Schematic representation of the genomic context adjacent to A1S_0143. (**B**) CP growth inhibition assays comparing wildtype with Δ*znuB*. Bacteria were cultured in CP growth media in the presence of increasing concentrations of CP with or without addition of 25 µM Zn, 25 µM Mn or both 25 µM Zn and 25 µM Mn. Data for 100 µg/ml CP are presented as the percent growth relative to bacterial growth without CP. Data are the average of three independent experiments with at least three biological replicates each. (**C**) Comparison of the effect of 25 µM TPEN on inhibition of wildtype and Δ*znuB* with or without addition of excess (50 µM) Zn. (**D**) Complementation of the sensitivity of Δ*znuB* to 20 µM TPEN by providing a wildtype copy of *znuB in trans*. Data are representative of three independent experiments with at least three biological replicates in each. * *p*<0.05, ** *p*<0.01, *** *p*<0.001 as determined by one-way ANOVA. (**E–F**) Assessment of *znu* promoter activity in the presence of increasing concentrations of TPEN (E) or Zn (F) using a GFP reporter. GFP activity is expressed as relative fluorescence where the intensity of the signal is normalized to the optical density of the culture.

In one mutant, 4G7, the transposon disrupts A1S_0143, which is annotated as encoding the permease component of a previously unstudied ABC family Zn transporter. Consistent with a role for A1S_0143 in Zn transport, disruption of this gene by transposon integration leads to increased sensitivity to CP (**Figure 3c**). A1S_0143 encodes a protein with similarity to ZnuB, a permease involved in Zn uptake that is found in several other bacteria [40]–[43]. ZnuB is typically part of an ABC-family transporter where ZnuC is the cognate ATPase and ZnuA is the periplasmic substrate-binding protein [40]–[43]. A1S_0144 and A1S_0146 encode an ATPase and a periplasmic binding protein similar to ZnuC and ZnuA, respectively (**Figure 4a**). Based on these observations and data presented below, we designate A1S_0143 as *A. baumannii znuB.* Notably, the *znuABC* genes are present in all available sequenced *A. baumannii* genomes (data not shown). To eliminate the potential for polar effects on neighboring genes caused by transposon integration, we generated a targeted deletion mutant in which *znuB* was replaced by an in-frame copy of the kanamycin resistance gene, *aph* (Δ*znuB*). Δ*znuB* was used for all subsequent experiments.

Since CP chelates both Mn and Zn, we first determined whether Δ*znuB* is more sensitive to Mn or Zn chelation by CP. CP growth inhibition assays were repeated with Δ*znuB* in CP growth medium. CP growth medium was chosen over the transposon screening medium since this medium allows for better titration of the Zn and Mn concentrations allowing for differentiation between the effects of Zn- and Mn-chelation on Δ*znuB.* Wildtype and Δ*znuB* exhibited similar growth levels and were not inhibited by CP when either Zn alone or Zn and Mn are added back to CP growth media (**Figure 4b** **and Figure S3**). In contrast, when only Mn was added back to CP growth media, CP significantly inhibited the growth of Δ*znuB*. The observation that growth of Δ*znuB* cannot be rescued by supplementation with Mn alone confirms that this mutant is sensitive to Zn limitation by CP. It was noted that even in the absence of CP, both wildtype and Δ*znuB* exhibited reduced growth in media alone compared to media supplemented with Mn and Zn (**Figure S4**). This suggests that the basal Mn and Zn levels in the media are insufficient to support growth of wildtype above that of Δ*znuB*, regardless of CP exposure. Consistent with this hypothesis, Δ*znuB* exhibited impaired growth compared to wildtype in rich media (LB) in the presence of the Zn-selective chelator, TPEN (**Figure 4c**). Moreover, the growth defect of the mutant was completely rescued by the addition of excess Zn, further supporting the role for ZnuB in Zn acquisition (**Figure 4c**). Finally, providing a full-length copy of *znuB in trans* rescues the TPEN sensitivity of Δ*znuB*, allowing growth at nearly wildtype levels (**Figure 4d**).

### Regulation of *znuB* in Zn-limiting conditions

The expression of bacterial high affinity transition metal transporters must be tightly regulated since transition metals can be toxic at high levels. Repression of metal uptake systems under nutrient replete conditions is frequently accomplished through metal-responsive transcriptional regulators like those in the Fur (ferric uptake regulator) family of transcriptional regulators. These regulators bind to target DNA sequences when bound to their cognate metal ligand to repress transcription. To determine whether *znuB* is under similar transcriptional control in *A. baumannii,* we first cloned the promoter for the *znu* operon upstream of a gene encoding GFP. Both wildtype and Δ*znuB* were then transformed with the plasmid carrying the *znu* reporter (p*znu*GFP) and grown in LB with or without the addition of TPEN or Zn. Wildtype bacteria exhibited increased fluorescence upon increasing concentrations of TPEN (**Figure 4e**). Similarly, Δ*znuB* demonstrated increasing fluorescence with increasing Zn chelation, although the basal level of GFP expression at all TPEN concentrations was higher in Δ*znuB* as compared to wildtype. Moreover, Δ*znuB* exhibited a greater increase in fluorescence at high concentrations of TPEN. Importantly, with the addition of excess Zn, the fluorescence observed for Δ*znuB* could be restored to levels equivalent to the basal levels observed in wildtype bacteria (**Figure 4f**). These results establish that the *znu* operon is up-regulated in Zn-limiting conditions and that Δ*znuB* experiences Zn starvation at higher Zn concentrations than wildtype.

**Figure 5:**
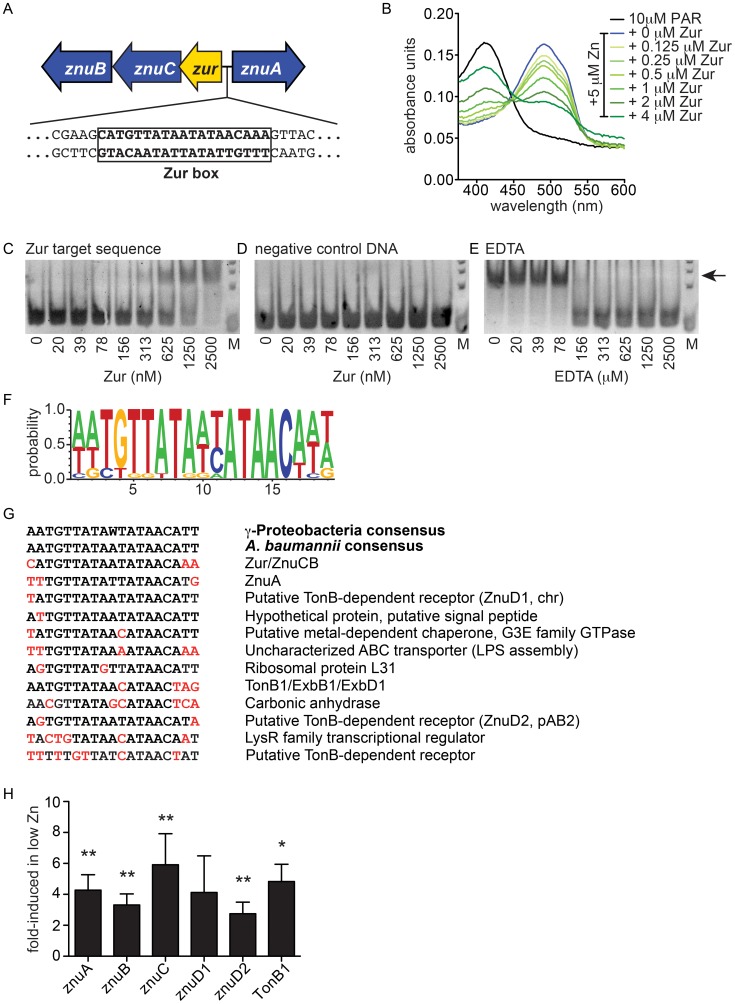
Genetic characterization of a putative Zn uptake system in *A. baumannii.* (**A**) Schematic representation of the genomic locus containing *znuA* and the *zurznuCB* operon. The sequence corresponding to a putative zur box is shown. (**B**) PAR assay measuring Zn binding by Zur. Spectral scans of solutions containing 10 µM PAR without Zn (black line), with Zn (blue line) or with Zn and increasing concentrations of Zur (green lines) are shown. (**C–D**) EMSA assays measuring binding of Zur to Zur target DNA (C) or negative control DNA (D) in the presence of Zn. (**E**) EMSA assay demonstrating loss of Zur binding to target DNA in the presence of increasing concentrations of EDTA. Arrow indicates mobility of shifted band. (**F**) Consensus sequence for the *A. baumannii* Zur box. (**G**) Predicted Zur-regulated genes and their predicted Zur boxes. (**H**) Expression analyses of genes predicted to be involved in Zn uptake across the outer (*znuD1*, *znuD2*, *TonB1*) and inner (*znuA*, *znuB*, *znuC*) membranes. Asterisks indicate relative expression that is statistically greater than one, where 1-fold indicates no change in expression in TPEN treated bacteria compared to untreated bacteria. * *p*<0.05, ** *p*<0.01 as determined by one sample *t* test using a reference value of one.

A potential mechanistic explanation for the Zn-regulated nature of *znu* was provided by the discovery of a putative Zur (Zinc uptake regulator) orthologue neighboring these genes (**Figure 5a**). Based on data described below, we refer to this gene as the *A. baumannii zur.* Zur is a member of the Fur family of transcriptional regulators and in other bacteria Zur mediates Zn-dependent repression of a number of genes including Zn uptake systems [40], [41], [44]–[48]. To determine if the *A. baumannii* Zur binds Zn, we performed a 4-(2-pyridylazo)-resorcinol (PAR) assay to measure Zur-mediated Zn binding. This assay is based on the principle that PAR exhibits a peak absorbance at approximately 410 nm in its free form, while this peak shifts to 495 nm upon Zn binding. Addition of a Zn binding protein to PAR-Zn leads to a reduction in the peak at 495 nm and an increase in the peak at 410 nm. When increasing concentrations of purified Zur were added to PAR-Zn, the Zn-bound peak at 495 nm decreased while the peak at 410 nm increased, establishing that *A. baumannii* Zur binds to Zn *in vitro* (**Figure 5b**).

Zur is a Zn-dependent repressor that binds to a 19 bp consensus sequence in the presence of Zn to repress transcription of a number of genes including those involved in Zn uptake [45]–[49]. The intragenic region between *znuA* and *zur* was searched for a possible Zur-binding site using the consensus sequence from γ-Proteobacteria [48]. This search yielded a possible Zur-binding sequence within 100 bp of the *znuA* translational start site (**Figure 5a**). Since a second candidate Zur binding site was not identified in the region between *znuA* and *zur,* it is likely that binding at this sequence leads to repression of both *znuA* and *zurznuCB*. To determine if Zur binds to this putative Zur box in a Zn-dependent manner, electrophoretic mobility shift assays were performed using purified Zur and oligonucleotides corresponding to the sequences shown in **Figure 5a**. When Zur is mixed with target DNA in the presence of excess Zn, the target DNA exhibits a shift in molecular weight consistent with Zur binding (**Figure 5c**). This shift is not observed when DNA corresponding to an intragenic region of *znuA* is used (**Figure 5d**). Moreover, addition of EDTA eliminated Zur binding to the target DNA, demonstrating that DNA binding is Zn-dependent (**Figure 5e**). Taken together, these data demonstrate that Zur is a Zn-binding protein that binds to the promoter of *znuA* and *zurznuCB* in a Zn-dependent manner.

Using the putative *A. baumannii* zur box sequence, we next conducted a BLAST search against the *A. baumannii* genome in order to identify additional candidate Zur-regulated genes (**Figure 5f** and **Table 2**). These putative Zur-regulated genes were further validated by searching for sequence motifs within their 5′ flanking sequences. The latter analysis, which employed the MEME analysis tool, independently identified the Zur box in a majority of the genes analyzed. Based on these results a list of putative Zur-regulated genes was constructed (**Table 2**). A number of the predicted Zur-regulated genes in *A. baumannii* are regulated by Zur in other bacteria. These include the ribosomal L31 protein, a carbonic anhydrase and the Znu system [44], [47], [48], [50] (**Figure 5g**). While the functional significance of Zur regulation has been elucidated for some of these genes, in many cases the function of the encoded proteins and their roles in Zn homeostasis have not been defined. Interestingly, *A. baumannii* Ab17978 encodes two predicted TonB-dependent receptors with highly conserved Zur-binding sequences located within their predicted promoters. One of these genes, A1S_2892 is encoded within the chromosome, while A1S_3475 is encoded within one of this strain's native plasmids. Respectively, these transporters are 41–42 percent identical and 58–61 percent similar, to ZnuD, a Zur-regulated receptor involved in Zn and heme transport in *N. meningitidis* [51]. Based on this sequence conservation and putative Zur-regulation, we have designated these genes as *A. baumannii znuD1* (A1S_2892) and *znuD2* (A1S_3475). A third TonB-dependent receptor was also identified; however, the predicted protein does not share significant sequence conservation with ZnuD.

**Table 2:** Locus tags and descriptions of predicted Zur-regulated genes.

Locus tag	Description^1^	E value^2^
A1S_0145	Zur (ZnuB, ZnuC)	6.19e-08
A1S_0146	ZnuA	9.90e-08
A1S_0391	LSU ribosomal protein L31p	5.74e-07
A1S_0410	LysR family transcriptional regulator	3.12e-06
A1S_0452	TonB (ExbB, ExbD)	1.17e-07
A1S_1679	Hypothetical protein	1.42e-05
A1S_2829	Putative TonB-dependent receptor	1.84e-05
A1S_2892	Putative TonB-dependent receptor, ZnuD1 (Pld)	6.19e-08
A1S_3103	Uncharacterized ABC transporter (possible LPS assembly operon)	5.74e-07
A1S_3225	Carbonic anhydrase	8.83e-07
A1S_3411	Putative metal chaperone involved in Zn homeostasis	1.24e-08
A1S_3412	Hypothetical protein, putative signal peptide	9.90e-08
A1S_3475	Putative TonB-dependent receptor protein, ZnuD2 (plasmid pAB2)	9.90e-08

1 Description of the predicted protein encoded at the indicated locus. If additional genes are predicted to be part of the same transcriptional unit, their encoded proteins are indicated in parentheses.

2 Expect values for the zur box motif.

To determine whether these candidate Zur-regulated genes are expressed under Zn-limiting conditions, *A. baumannii* was grown in LB or LB supplemented with the Zn-chelator, TPEN, at a concentration of 10 µM. This concentration was selected because at this concentration of TPEN Δ*znuB* begins to exhibit decreased growth compared to wildtype. This suggests that the Znu system is required for optimal growth at 10 µM TPEN and is therefore likely to be induced under these conditions in wildtype bacteria. Consistent with this hypothesis, transcripts for *znuA, znuB, znuC, znuD1* and *znuD2* were all increased in TPEN-containing media compared to media without TPEN (**Figure 5h**). Up-regulation of *znuABC*, *znuD1*, and *znuD2* under Zn-limiting conditions supports the hypothesis that these genes are involved in Zn acquisition.

The outer membrane of Gram-negative bacteria represents a significant permeability barrier for ions and small molecules. In many bacteria, transport of transition metal ions across the outer membrane is thought to occur by diffusion through non-selective porins. However, the expression of two TonB-dependent receptors in *A. baumannii* under Zn-limiting conditions suggests that transport of Zn across the outer membrane of this organism may be an energy-dependent process similar to the case in *N. meningitidis* [51], [52]. Transport through TonB-dependent receptors requires the TonB-ExbB-ExbD system, which harnesses energy from the proton motive force generated at the inner membrane to facilitate transport across the outer membrane. Interestingly, *A. baumannii* encodes two predicted TonB-ExbB-ExbD systems. One of these systems appears to be under transcriptional control by Zur, based on the presence of a Zur-binding consensus sequence within the *tonB* promoter (**Figure 5g**). We have designated the putative Zur-regulated system TonB1-ExbB1-ExbD1, based on its location within the chromosome relative to the position of the predicted Fur-regulated system. Although TonB1-ExbB1-ExbD1 was previously shown to be up-regulated in the presence of the iron chelator 2,2-dipyridyl, we sought to determine whether Zn-limiting conditions induce expression of the *tonB1exbB1exbD1* operon [53]. In the presence of 10 µM TPEN, the *tonB* gene is up-regulated 4- to 5-fold supporting a model whereby translocation of Zn across the outer membrane is an energy-dependent process in *A. baumannii* (**Figure 5h**).

### ZnuB contributes to the pathogenesis of *A. baumannii* pulmonary infections

**Figure 6:**
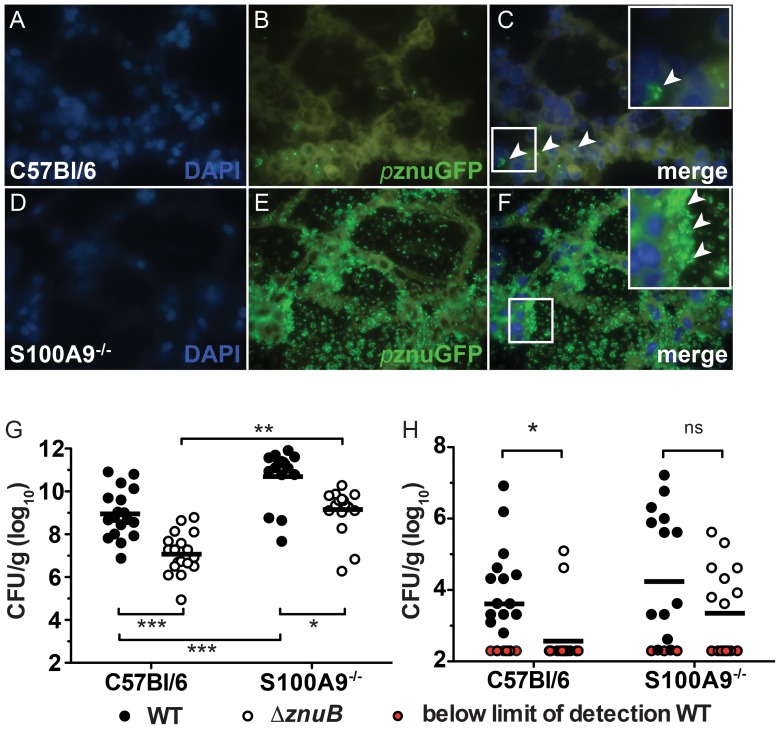
Contribution of the Znu system to pathogenesis *in vivo*. (**A–F**) *In vivo* GFP expression driven by the *znu* promoter. Frozen sections of lungs harvested at 36 hpi from wildtype (A–C) or S100A9^−/−^ (D–F) mice infected with WT::*p*znuGFP and stained with DAPI. Arrowheads in the merged images indicate bacteria expressing GFP. (**G–H**) Competition infection between wildtype and Δ*znuB*. Bacterial burden in lungs (**G**) and livers (**H**) of mice co-infected with wildtype *A. baumannii* and Δ*znuB*. Red symbols indicate CFU below the limit of detection. * *p*<0.05, ** *p*<0.01, *** *p*<0.001 as determined by one-way ANOVA.

To define the contribution of the Znu system to pulmonary infections, we first examined *znu* promoter activity in both wildtype and S100A9^−/−^ mice infected with WT::p*znu*GFP. In both wildtype and S100A9^−/−^ mice we observed strong promoter activity as evidenced by GFP signal within lungs harvested at 36 hpi (**Figure 6a–f** **and Figure S5**). Although in both wildtype and S100A9^−/−^ mice we detected strong GFP signal, the overall increased bacterial burden in S100A9^−/−^ mice led to greater total signal in these sections. These data demonstrate that the *znu* genes are expressed *in vivo* and that *A. baumannii* is Zn starved in the lung. Furthermore, these data imply that even in the absence of CP, the host sequesters Zn away from *A. baumannii* suggesting the presence of additional mechanisms for Zn withholding in the vertebrate lung.

To determine the direct contribution of ZnuB to *A. baumannii* pathogenesis, wildtype and S100A9^−/−^ mice were co-infected with an equal mixture of wildtype and Δ*znuB* and bacterial burdens were quantified at 36 hpi in lungs and livers. Wildtype *A. baumannii* significantly outcompetes Δ*znuB* for colonization of the lungs of both wildtype and CP-deficient mice (**Figure 6g**). Notably, Δ*znuB* was only detected in the livers of two mice (10 percent) following intranasal infection (**Figure 6h** **and Figure S6**). Moreover, dissemination of Δ*znuB* to the liver is partially rescued in S100A9^−/−^ mice. A similar competition infection comparing wildtype *A. baumannii* to a strain containing a kanamycin resistant cassette in an unrelated gene did not reveal a similar defect, demonstrating that the presence of the kanamycin marker alone is not responsible for the observed phenotype (data not shown). These data suggest that CP-mediated Zn-chelation is critical to limit bacterial dissemination from the primary site of lung infection. Taken together, the results of the *in vivo* studies demonstrate that Zn acquisition through ZnuB contributes to *A. baumannii* pathogenesis. Notably, no significant difference was observed when comparing wildtype and Δ*znuB* in mono-infections (**Figure S6B–C**).

### Zn chelation reverses carbapenem resistance in MDR *A. baumannii*

**Figure 7:**
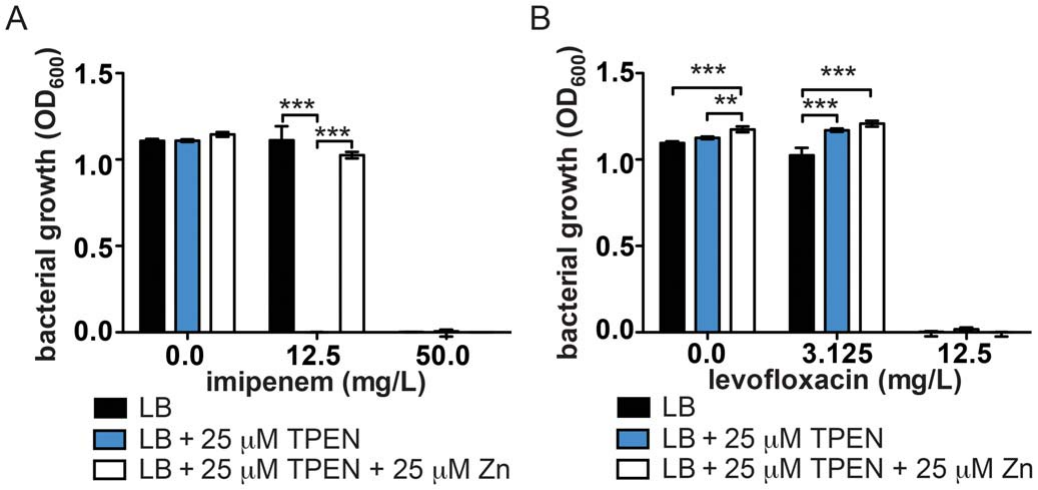
Effect of Zn chelation on resistance to imipenem and levofloxacin in an MDR isolate of *A. baumannii.* Bacterial growth measured at 24 hours in LB medium in the presence of increasing concentrations of imipenem (**A**) or levofloxacin (**B**) and 25 µM TPEN, with or without addition of excess Zn. Data for concentrations near and above the clinical breakpoints for resistance are shown.

One of the few remaining antibiotic classes available for the treatment of *A. baumannii* infections is the carbapenems. However, carbapenem resistance is becoming increasingly common primarily through dissemination of genes encoding carbapenem hydrolyzing enzymes or carbapenemases [54], [55]. Interestingly, many of these carbapenemases are metalloenzymes that require Zn for their hydrolyzing activity [56], [57]. This raises the possibility that Zn limitation may serve as a valuable adjunct to carbapenem therapy by decreasing carbapenemase activity. To test this hypothesis, we determined the imipenem MIC against the carbapenem resistant clinical isolate, AB0057, in the presence or absence of 25 µM TPEN. Treatment with TPEN reduces the imipenem MIC to below the clinical breakpoint for imipenem resistance in *A. baumannii* and this effect is reversed by addition of excess Zn (**Figure 7a**). As a control, these experiments were performed with the fluoroquinolone antibiotic, levofloxacin. In AB0057, levofloxacin resistance is mediated by mutations within *gyrA* (DNA topoisomerase IIA) and *parC* (DNA topoisomerase IV) and therefore should not be reversed by Zn chelation [58]. The levofloxacin MIC was either unchanged or slightly increased by Zn limitation and remained above the breakpoint for clinically defined resistance (**Figure 7b**). These results highlight Zn limitation as a possible mechanism to combat carbapenem resistance in *A. baumannii*.

## Discussion

Transition metals occupy an essential niche within biological systems. The essentiality of transition metals to invading bacterial pathogens has been exploited by vertebrate hosts as an innate defense strategy. Most work in the area of nutritional immunity has focused on iron sequestration as a mechanism of host defense; however, it is now appreciated that nutritional immunity includes strategies to withhold other essential metals such as Mn and Zn [17], [59]. We have demonstrated that Mn and Zn chelation by CP inhibits *A. baumannii* growth *in vitro.* We have also determined that CP is abundant in the lungs of *A. baumannii*-infected mice where it contributes to defense against *A. baumannii* pulmonary infections. Moreover, we have used CP to elucidate physiological processes that are disrupted by Mn and Zn chelation by screening a transposon library for mutants with increased or decreased susceptibility to CP. Using this screen we identified a Zn acquisition system in *A. baumannii* and defined its Zn-dependent regulation and its roles in Zn uptake and pathogenesis.

As summarized above, utilizing CP as a probe has uncovered the ZnuABC Zn uptake system in *A. baumannii*. A targeted deletion of *znuB* is more sensitive to CP and other Zn chelators, particularly in the presence of excess Mn. Notably, the phenotype of the *znuB* mutant can be rescued by addition of excess Zn, suggesting that *A. baumannii* possesses an additional mechanism to transport Zn across the inner membrane. Although we did not identify additional inorganic ion transporters in the analysis of the putative Zur regulon, it is possible that Zn is transported through a low affinity or non-specific transporter. Based on the observation that excess Mn potentiates the effects of Zn chelation on Δ*znuB*, it is also possible that Zn acquisition in this mutant depends on an inner membrane Mn transporter. In this case, the presence of excess Mn would occupy Mn transport systems, further reducing Zn acquisition by Δ*znuB*.

Elucidation of the *A. baumannii* Zur regulon identified genes encoding two putative ZnuD orthologues and a TonB system. These genes were all up-regulated in Zn-limiting conditions. Although it was previously thought that transition metal ions such as Zn^2+^ and Mn^2+^ freely diffuse through non-selective porins, the identification of ZnuD in *Neisseria spp.* suggests that in some bacteria, transport of Zn across the outer membrane may be an energy-dependent process [51]. *A. baumannii* strain 17978 has two putative ZnuD orthologues, one encoded on the chromosome and a second on a plasmid. Moreover, *A. baumannii* encodes a TonB/ExbB/ExbD system that is up-regulated in low Zn conditions suggesting that this energy-generating system may be dedicated to Zn import. An energy generating system that is activated upon Zn starvation provides compelling evidence for an energy-dependent mechanism of Zn transport across the outer membrane. However, it is not yet known whether ZnuD transports free or chelated Zn. ZnuD from *N. meningitidis* contributes to heme acquisition when expressed in *E. coli* [52]. This finding suggests that ZnuD is either a heme transporter that is up-regulated in low Zn conditions, or that ZnuD transports more than one substrate. If the latter is true, ZnuD may transport Zn in a chelated form analogous to the siderophore systems used to transport iron. It remains to be determined whether either of the ZnuD transporters in *A. baumannii* directly transports Zn or heme.

In addition to the role for ZnuB in Zn acquisition and resistance to CP *in vitro,* this protein also contributes to pathogenesis *in vivo.* Specifically, co-infection with wildtype demonstrates that Δ*znuB* is less able to compete with wildtype *in vivo.* Notably, in the lung, Δ*znuB* exhibits reduced competitive fitness in both wildtype and CP-deficient mice, suggesting that other factors in addition to CP may contribute to Zn limitation within the lung. In contrast, the ability of Δ*znuB* to disseminate past the primary site of infection was almost completely abrogated during co-infection with wildtype *A. baumannii* in wildtype mice. However, in CP-deficient mice, Δ*znuB* disseminated efficiently to the liver where bacterial burdens for the mutant approached those for wildtype bacteria. These data suggest that while CP is an important contributor to innate immunity in the lung, CP may be more important in defending against dissemination to secondary sites like the liver. Previous studies have demonstrated an important role for CP in limiting bacterial replication and abscess formation in the liver during *S. aureus* systemic infections [20]. This finding implicates CP as being critical to limit replication in the liver. However, it remains to be determined for *A. baumannii* infections whether CP inhibits bacterial survival in the bloodstream or whether CP is important for protecting against colonization of the liver itself.

Vertebrate hosts have evolved elegant mechanisms to withhold essential metals from invading pathogens. Likewise, bacterial pathogens have evolved efficient means to acquire metals from their vertebrate hosts. Based on these important roles for transition metals at the pathogen-host interface, bacterial metal acquisition systems represent possible targets for therapeutic development. The observation that Zn limitation reverses imipenem resistance in a carbapenemase-producing clinical isolate suggests that Zn chelation itself or therapies directed against bacterial Zn acquisition systems may be viable strategies to use in combination with existing antimicrobial compounds. Given the dire need for new antibiotics effective against MDR *A. baumannii*, combination strategies like these may be critical in battling this important public health threat.

## Materials and Methods

### Ethics statement

All experiments involving animals were reviewed and approved by the Institutional Animal Care and Use Committee of Vanderbilt University. All experiments were performed according to NIH guidelines, the Animal Welfare Act, and US Federal law.

### Bacterial strains and reagents

All experiments were performed using *A. baumannii* strain ATCC 17978 (Ab17978) or its derivatives unless otherwise noted. The transposon mutant library was constructed using the EZ-Tn5 transposome system (Epicentre) as described previously [60]. *A. baumannii* strain AB0057 was a gift from Robert Bonomo and is a carbapenemase-producing strain with an imipenem MIC of >12 µg/ml [58]. Chemicals and antibiotics were purchased from Sigma unless otherwise noted. Recombinant human calprotectin was used for all *in vitro* experiments and was expressed and purified as described previously [20]. All experiments involving liquid cultures were performed at 37°C with shaking at 180 rpm unless otherwise specified.

### *In vitro* growth inhibition assays with calprotectin

Overnight cultures of *A. baumannii* were diluted 1∶20 in Chelex-treated RPMI (ChxRPMI) without metals added back and incubated for 1 hour at 37°C. Following incubation, the cultures were further diluted 1∶50 in CP growth media, which consists of 20 percent CP buffer (20 mM Tris, pH 7.5, 100 mM NaCl, 10 mM β-mercaptoethanol, 3 mM CaCl_2_), and 80 percent ChxRPMI to which was added 0.1 mM CaCl_2_, 1 mM MgSO_4_ and 10 µM FeSO_4_, with increasing concentrations of CP (0–400 µg/ml). In some assays, Mn and/or Zn were also added at the indicated concentrations. Bacteria were incubated at 37°C and growth was monitored at 1- to 2-hour intervals throughout the time course. Bacterial growth in the presence of CP was normalized to growth without CP and dose-response curves were generated by non-linear regression analyses. IC_50_ values were obtained from the best-fit curves of data obtained after 10–12 hours of culture.

### ICP-MS analyses of intracellular Zn concentrations

Bacteria were cultured overnight in ChxRPMI with 0.1 mM CaCl_2_, 1 mM MgSO_4_ and 10 µM FeSO_4_ added. Bacteria were sub-cultured 1∶20 in fresh ChxRPMI for 1 hour. The bacteria were then sub-cultured 1∶50 into 10 mL CP growth media with 0 or 37.5 µg/ml CP and 50 µM Mn and incubated for approximately 6 hours (OD_600_ = 0.6). Bacteria were then pelleted by centrifugation (10 minutes, 6,000 *g*), washed twice with water and transferred to Teflon vials. Bacterial pellets were dried by incubation at 50°C overnight then digested by boiling in nitric acid for 6 hours at 130°C.

Elemental quantification was performed on the Thermo Element 2 HR-ICPMS (Thermo Fisher Scientific, Bremen, Germany) coupled with ESI auto sampler (Elemental Scientific, Omaha, NE). The HR-ICPMS is equipped with a PFA microflow nebulizer (Elemental Scientific, Omaha, NE), a double channel spray chamber (at room temperature), a magnetic sector followed by an electric sector, and a second electron multiplier. The sample uptake was achieved through self-aspiration via 0.25 mm ID sample probe and sample capillary. The operation parameters are listed in **Table 3**.

**Table 3:** HR-ICP-MS parameters.

Instrument	Element 2 HR-IC-MS
RF power	1200 W
Cool gas	16.00 L min^−1^
Auxiliary gas	0.8 L min^−1^
Sample gas	1.05 L min^−1^
Resolution mode	Medium resolution (4000)
Isotopes measured	^55^Mn, ^57^Fe, ^63^Cu, ^65^Cu, ^66^Zn, ^68^Zn
Runs	10
Passes	1
Samples per peak	20
Sample time	0.01 s

Results were normalized to the dry weight of the sample measured prior to analysis. Quantities of Mn, Zn and Fe were then normalized to Cu as an internal reference since Cu levels were not altered by CP treatment (data not shown).

### *A. baumannii* infections

Wildtype C57BL/6 mice were obtained from Jackson Laboratories. S100A9^−/−^ mice were a gift from Wolfgang Nacken (Institute of Experimental Dermatology, University of Münster, 48149 Münster, Germany). All of the infection experiments were approved by the Vanderbilt University Institutional Animal Care and Use Committee. *In vivo* studies utilized the murine model of *A. baumannii* pneumonia previously developed in our laboratory with a few modifications [60]. Briefly, mice were infected with 3–5×10^8^ CFU *A. baumannii* in 50 µl PBS. At the indicated times post infection mice were euthanized and CFU were enumerated in lungs and livers following tissue homogenization and plating on bacterial growth medium. We initially performed time course studies comparing male C57BL/6 mice with S100A9^−/−^ mice for their susceptibilities to *A. baumannii* infection to determine the optimal age at which to perform experiments (data not shown). Based on these studies, 9–10 week-old male mice were used for subsequent experiments. For infections comparing wildtype and Δ*znuB,* bacteria were cultured in RPMI instead of LB. For co-infections, equal numbers of wildtype and Δ*znuB* were mixed to yield a total of 1×10^10^ CFU/ml and mice were infected with 50 µl of the combined mixture. At 36 or 72 hours post infection (hpi), mice were euthanized and differential bacterial counts were determined in lungs and livers by plating organ homogenates on LB agar or LB agar supplemented with 40 µg/ml kanamycin. To calculate the competitive index, the ratio of Δ*znuB* to wildtype bacteria recovered from lungs and livers was divided by the input ratio. When no bacteria (either wildtype, Δ*znuB* or both) were recovered, the number of recovered bacteria was set at the limit of detection for the assay (approximately 600 CFU/g for lungs and 200 CFU/g for livers). For histological analyses lungs were inflated with 1 ml of 10 percent formalin, fixed, embedded and stained as described previously [60]. For Imaging Mass Spectrometry and GFP reporter assays, mice were infected as described above with wildtype bacteria or WT::p*znu*GFP (described below). Upon sacrifice, lungs were perfused with a 1∶1 mixture of water and Optimal Cutting Temperature (OCT) compound (TissueTek). Lobes were marked using dye-based drawing ink, and snap frozen using a mixture of hexanes and dry ice. Organs were stored at −80°C until ready for use.

### Imaging mass spectrometry of infected and uninfected lungs

Trifluoroacetic acid (TFA) and Sinapinic Acid were purchased from Sigma-Aldrich Chemical Co. (St. Louis, MO, USA). HPLC-grade acetonitrile, Ammonium bicarbonate powder, and technical grade hexanes were purchased from Fisher Scientific (Pittsburgh, PA, USA). OCT was purchased from VWR International (Suwanee, GA, USA). Dye-based drawing ink was purchased from Higgins Ink (Chartpak Inc. Leeds, MA, USA). Phosphate Buffered Saline (10×) was purchased from Life Technologies Corporation (Grand Island, NY, USA).

Frozen lung tissue was cut into 10 µm thick sections at −20°C using a cryostat (Leica Microsystems, Bannockburn, IL, USA) and thaw mounted onto an indium-tin oxide (ITO) coated glass slide (Delta Technologies, Loveland, CO, USA). Mounted tissue sections were washed to remove excess lipids and salts using sequential chilled solutions of phosphate buffered saline, 100 mM ammonium bicarbonate, and 95 percent ethanol, all performed cold. Sinapinic acid matrix was prepared at 20 mg/ml in 1∶1 acetonitrile∶water with 0.1% TFA. Matrix was applied to tissue using a Portrait 630 (Labcyte, Sunnyvale, CA, USA) acoustic robotic microspotter. Matrix was applied in a block pattern with each spot being 200 µm in diameter and spaced 250 µm apart center to center, using three passes of fifteen 170 pL drops.

Spotted arrays were imaged using a Bruker Autoflex Speed time-of-flight mass spectrometer (Bruker Daltonics, Billerica, MA, USA) in linear positive–ion mode equipped with a SmartBeam laser (Nd:YAG, 355 nm). Detector voltage gain was set to 2910 volts. Spectra were collected at each spot as a sum of 400 laser shots in 50 shot steps. The laser repetition rate was 1,000 Hz, first and second ion source voltages were 19.47 kV and 17.82 kV respectively with a delay time of 350 ns and a lens voltage of 6 kV. Measured mass range was from 3,000–27,000 Daltons. Spectra were analyzed using Fleximaging 3.0 Software (Bruker Daltonics) and were normalized to total ion current. Ion density maps were extracted.

### Transposon mutant library screen

A transposon library was constructed using the EZ-Tn5 transposome system as described previously [60]. Since growth of wildtype bacteria is significantly attenuated in ChxRPMI without Mn or Zn added back, the growth conditions were modified for the primary screen in order to facilitate rapid screening of over 4000 mutants. Mutants were inoculated directly from frozen stocks into LB and incubated at 37°C overnight. Bacteria were then sub-cultured at a 1∶20 dilution in ChxRPMI for 1 hour at room temperature. Following the 1-hour subculture, bacteria were diluted 1∶50 into 96-well plates containing 100 µl of CP screening media, which consisted of 50 percent RPMI (not chelex-treated) without supplemental metals and 50 percent CP (40 µg/ml) in CP buffer. The plates were incubated at 37°C with shaking at 180 rpm for 10–12 hours, and growth was monitored by measuring the optical density of the cultures at 600 nm. Mutants whose growth in the presence of CP differed by more than two standard deviations from the plate average were selected for further analysis. The phenotypes of the selected mutants were confirmed by repeating the inhibition assay as described above for the primary screen in triplicate cultures. Only mutants that exhibited growth comparable to wildtype in media without CP but whose growth in the presence of CP differed significantly from that of wildtype bacteria were selected for insert identification. The transposon insertion sites were determined by inverse PCR or by sequencing directly from chromosomal DNA using primers KAN-1 (5′ - ACCTACAACAAAGCTCTCATCAACC - 3′) and KAN-2 (5′ - CTACCCTGTGGAACACCTACATCT - 3′).

### Construction of Δ*znuB*

Approximately 1000 bp of flanking DNA sequence was amplified from the immediate 5′ and 3′ regions surrounding *znuB.* The kanamycin resistance gene *aph* was amplified from pUCK1 and the three PCR products were stitched by overlap extension PCR as described previously [61]. This PCR product was cloned into pCR2.1 (Invitrogen) and sequence verified. The product was then re-amplified and the resulting linear DNA product was transformed into Ab17978 to generate an in-frame allelic replacement of *znuB* with *aph*. Transformants were selected on LB agar supplemented with 40 µg/ml kanamycin. The resulting colonies were screened for integration of the kanamycin cassette into the correct locus using locus-specific primers that anneal outside the region contained within the knock out construct.

### *In vitro* growth inhibition assays with TPEN

Overnight bacterial cultures were sub-cultured 1∶20 in fresh LB for 1 hour followed by a 1∶50 back dilution into LB containing various concentrations of tetrakis-(2-pyridylmethyl)ethylenediamine (TPEN) (0–100 µM) with or without addition of 100 µM ZnSO_4_. Bacterial growth was assessed over approximately 24 hours. Data from the 12-hour time point, which are representative of the trends in bacterial growth over the full time course, are shown. Data were averaged from at least three biological replicates and statistical significance was determined by one-way ANOVA.

### Complementation of the TPEN sensitivity of Δ*znuB*

A vector for complementation was constructed by first amplifying the region separating *znuA* and *zurznuCB* by PCR using primers ZnuPro1 (5′ – CCGAATTCGACAGACCATCAATTAGTAATAGC – 3′) and ZnuPro2 (5′ – CCGAATTCAAGAACTCATCATAGACATAACCTC – 3′). The resulting product was cloned into the EcoRI site of the *A. baumannii*: *E. coli* shuttle vector pWH1266 to generate p*znu*Pro. The open reading frame encoding *znuB* was PCR amplified from WT genomic DNA using primers znuBcompF2 (5′ – CCCGGATCCATGATGGAATGGTTACAATTATTG – 3′) and znuBcompR2 (5′ – CCCGTCGACGATTTAGGCTGCTTGAGTTTG – 3′), which include BamHI and SalI sites, respectively. The resulting product was cloned into the BamHI and SalI sites of p*znu*Pro to generate the p*znuB* vector used for complementation experiments. The empty vector, p*znu*Pro, and the complementation vector, p*znuB*, were each introduced into Δ*znuB* by electroporation. In addition, the empty vector was also transformed into wildtype bacteria to serve as an additional control. For growth assays, bacteria were grown overnight in LB+500 µg/ml ampicillin with aeration at 37°C, and the next morning, overnight bacterial cultures were sub-cultured 1∶50 in fresh LB+500 µg/ml ampicillin for an additional hour. These cultures were then re-seeded 1∶100 into LB+500 µg/ml ampicillin and containing various concentrations of TPEN (0–80 µM) on a 96-well plate. Bacterial growth was assessed by OD_600_ over 24 hours with aeration at 37°C. Data are presented from the 12-hour time point, which are representative of the trends in bacterial growth over the full time course. Statistical significance was determined by one-way ANOVA.

### Construction of a *znu* reporter and measurement of GFP expression

The gene encoding GFP was sub-cloned from pMU125 into the BamHI and SalI sites of p*znu*Pro to generate p*znu*GFP, which was subsequently introduced into wildtype and Δ*znuB* by electroporation. Empty pWH1266 was included as a control. The resulting strains, designated as WT::pWH1266, WT::p*znu*GFP, and Δ*znuB*::p*znu*GFP were cultured in LB with increasing concentrations of TPEN as described above or with increasing concentrations of Zn (0–1000 µM) and GFP fluorescence was measure using a BioTeq plate reader with excitation and emission filters set for 395 and 509 nm, respectively. GFP fluorescence was normalized to bacterial growth, and data from approximately 9 hours of growth are shown. Data were averaged from three biological replicates.

### Identification of a putative Zur-binding consensus sequence and Zur-regulated genes

The intergenic region between *znuA* and *zur* was searched for possible Zur binding motifs using the consensus 19 bp zur box (AATGTTATAWTATAACATT) derived from the analysis of 13 genes from several gamma-Proteobacteria [48]. BLAST analysis was then employed to search for this *A. baumannii* zur box in the Ab17978 genome. Once possible Zur-regulated genes were identified, 5′ flanking sequences from each gene were searched for motifs using the Multiple Em for Motif Elicitation (MEME) tool [62]. The search parameters were limited to search for motifs on the forward strand only and to motifs ranging from 12 to 50 nucleotides in length. This search independently identified the putative zur box motifs from the majority of genes analyzed. Each of the zur box sequences was then entered into WebLogo 2.0 to generate a sequence logo that graphically represents the degree of sequence conservation at each nucleotide position [63].

### Zur expression and purification

*A. baumannii zur* was PCR amplified using primers 167×2 (5′- CCGCTCGAGTTAGCGAGCTGTGCGG-3′) and 167×4 (5′-CCGCTCGAGGTTATGTCTATGATGAGTTCTTGC-3′) and cloned into pET15b (Novagen) in frame with the N-terminal 6xHis tag. *E. coli* BL21(DE3) cells were transformed with the Zur expression vector and grown at 37°C to an OD_600_ of 0.6 before induction with 0.5 mM IPTG. Following induction, bacteria were maintained at 37°C for an additional 6 hours. Cells were harvested by centrifugation at 6000 *g* and resuspended in lysis buffer (50 mM NaH_2_PO_4_, 300 mM NaCl, 20 mM imidazole, 1 mg/ml lysozyme). Cells were lysed by five passes through an EmulsiFlex homogenizer (Aventin, Inc., Ottawa, ON) at 20,000 psi. Following disruption the lysates were centrifuged at 8,000×*g* to remove unlysed cells and debris. Insoluble material was separated by ultracentrifugation at 100,000×*g* for 1 hour at 4°C. The resulting supernatants were applied to a Ni: NTA column pre-equilibrated with lysis buffer. The column was washed with approximately 20 bed volumes of wash buffer (50 mM NaH_2_PO_4_, 300 mM NaCl, 25 mM imidazole) followed by sequential washes with increasing concentrations of imidazole (50 mM, 100 mM, 150 mM, 175 mM, 200 mM, 225 mM and 250 mM). Zur eluted free from contaminating proteins in buffer containing between 200 and 250 mM imidazole. CaCl_2_ was added to the eluted sample to a final concentration of 2.5 mM. Removal of the His tag was achieved by thrombin cleavage. Briefly, two units of restriction grade thrombin (Novagen) were added and the sample was cleaved and dialyzed overnight at 4°C into buffer containing 20 mM Tris-HCl, pH 8.0, 150 mM NaCl and 2.5 mM CaCl_2_.

### PAR assay for Zn binding

Zn binding by Zur was determined using the Zn-binding dye PAR. Free PAR exhibits a peak absorbance at approximately 410 nm, which shifts to 500 nm upon Zn binding. To determine if Zur binds Zn, increasing concentrations (0 µM–4 µM) of purified Zur were added to solutions containing 10 µM PAR and 5 µM Zn in 50 mM HEPES, pH 8.0. Spectra were obtained for free PAR and PAR bound to Zn as controls, which were compared to the spectra obtained in the presence of Zur. A decrease in the absorbance at 500 nm, together with an increase in the absorbance at 410 nm indicates binding of Zn by Zur.

### Electrophoretic mobility shift assays

EMSA probes were generated from primers zur1 (5′ – CGAAGCATGTTATAATATAACAAAGTTAC – 3′) and zur2 (5′-GTAACTTTGTTATATTATAACATGCTTCG – 3′) for the *znuA/zurznuCB* Zur box or zurneg1 (5′ – GTTACAAAAATACCTAAGATTAATCGAAT – 3′) and zurneg2 (5′ – ATTCGATTAATCTTAGGTATTTTTGTAAC – 3′) for the intragenic sequence from *znuA* that served as a negative control. Each primer was incubated at 65°C for 10 min then incubated in a water bath that was allowed to slowly equilibrate from 65°C to room temperature. Increasing concentrations of purified Zur (0–2500 nM) were incubated with 250 nM of the probes described above in EMSA buffer (20 mM Tris-HCl, pH 8.0, 50 mM KCl, 1 mM DTT, 5% glycerol and 100 µM ZnCl_2_) for 20 min at room temperature. Following incubation, 20 µl of each sample was loaded onto 4% polyacrylamide gels and electrophoresed for 30 min at 100 V. The gels were stained with SYBR green (Invitrogen) and visualized on an Alpha Imager gel documentation system using UV light. In order to test whether Zn chelation could eliminate Zur binding to target DNA, 250 nM of Zur target DNA was incubated with 1250 nM Zur in EMSA buffer with increasing concentrations of EDTA (0–2500 µM). EDTA was chosen as a chelator for these assays since it has higher solubility in water than TPEN.

### Expression analyses

*A. baumannii* was grown in LB or LB supplemented with 10 µM TPEN for approximately 6 hours at 37°C with shaking at 180 rpm. This concentration was selected because at this concentration of TPEN Δ*znuB* begins to exhibit decreased growth compared to wildtype bacteria. This suggests that the Znu system is required for optimal growth under these conditions and is therefore likely to be induced under these conditions in wildtype bacteria. Reverse transcription was carried out on 2 µg total RNA using 200 units M-MLV reverse transcriptase and 1 µg random hexamers according to the manufacturer's protocol (Promega, Madison, WI). PCR was performed using 10 ng cDNA template (0.01 ng template for 16S rRNA). Control reactions were performed on RNA samples without reverse transcriptase treatment. These reactions did not yield detectable signal. Sequences for the primers used in these analyses are shown in Table 4. Data were analyzed from at least three biological replicates after normalizing to 16S rRNA. Data from TPEN treated samples are presented as the fold-induction relative to untreated samples.

**Table 4:** Primers used for qPCR.

Target gene	Primer sequences
*znuA*	GAGTACGTTAGGTTGGAGTCAGG
	GGTCATCCGTTAAGGCACC
*znuB*	TGGCACATGGAACCTTACTTG
	AACACTGACACCGAGTTGAGC
*znuC*	CATCTCATAGTCTTCCTTTACGG
	TGTGAGGTCTGCTATTGTGAGG
*znuD1*	CAAGTTGCATTACGTGTTGAGG
	TATGGACTAACTCAATACGCCC
*znuD2*	AATATGGAGGTGGAGCATCTG
	GCGTACCTAATCTTGACTCTGC
*tonB1*	GTCATCACGGCACTAATTGCG
	GACTTGGAATACCGCTGCTACG

### *In vivo* GFP reporter activity

Frozen lungs of mice infected with wildtype or WT::p*znu*GFP were sectioned as described above and stained with DAPI (0.01 ng/ml) for 30 minutes at room temperature. Sections were washed with PBS, and coverslips were sealed over the sections using clear nail polish. The sections were visualized using an Olympus BX60 microscope. Images were captured with an Olympus DP71 camera using DP Controller and analyzed using DP Manager software.

### Imipenem and levofloxacin inhibition assays

Bacteria were cultured in LB overnight then sub-cultured 1∶1000 in LB containing 25 µM TPEN and either imipenem (0–20 µg/ml) or levofloxacin (0–25 µg/ml). Where indicated, ZnSO_4_ was added to a final concentration of 25 µM. Bacteria were cultured for 24 hours while the optical densities of the cultures were monitored at 600 nm. Minimum inhibitory concentrations were determined as the concentration in the first well in which no bacterial growth was observed. MICs were comparable for each of the antibiotics in LB as compared to Mueller Hinton Broth (data not shown).

## Supporting Information

Figure S1 *A. baumannii* growth in the presence of increasing concentrations of CP (solid lines) or ΔSI/SII, a variant of CP that no longer binds Mn and Zn (dashed lines). Black indicates no Mn or Zn added, while orange indicates 25 µM Mn and 25 µM Zn added back. Data represent the average of three biological replicates.
(TIF)

Figure S2 Bacterial burdens in lungs of wildtype and S100A9^−/−^ mice harvested at 72 hpi with *A. baumannii*. Each symbol represents one animal. Only data from mice surviving to 72 hours are shown. The data were combined from two independent experiments with at least 10 mice per experiment per genotype.
(TIF)

Figure S3 CP growth inhibition assays comparing wildtype with Δ*znuB*. Bacteria were cultured in CP growth media in the presence of increasing concentrations of CP without supplementation (A) or with addition of 25 µM Zn (B), 25 µM Mn (C) or both 25 µM Zn and 25 µM Mn (D). Data are presented as the percent growth relative to bacterial growth without CP. Curve fit was performed using a non-linear regression with variable slope. Curves are not drawn for WT+Mn and WT+Zn and Mn because these data are not converged and therefore the same curve fit parameters could not be used. Data are the average of three independent experiments with at least three biological replicates each.
(TIF)

Figure S4 Growth curve analyses of wildtype and Δ*znuB* cultured in CP growth media with (orange) or without (black) added Mn and Zn. Data are averaged from at least three biological replicates. Error bars, which may be obscured by the symbols in some cases, represent one standard deviation from the mean.
(TIF)

Figure S5 *In vivo* GFP expression driven by the *znu* promoter. Frozen sections of lungs harvested at 36 hpi from wildtype (A–D) or S100A9^−/−^ (E–H) mice infected with WT::p*znu*GFP and stained with DAPI. Sections from lungs of mice infected with wildtype bacteria without the p*znu*GFP plasmid (I–L) are shown for comparison.
(TIF)

Figure S6 (**A**) Competitive indices of Δ*znuB* compared to wildtype *A. baumannii* in lungs and livers of wildtype and S100A9^−/−^ mice. Each symbol represents one animal. Red symbols indicate mice in which the competitive index was determined by setting the recovered CFU for Δ*znuB* at the limit of detection since there were no recoverable CFU of Δ*znuB* in these mice. (**B–C**) Bacterial burdens from monoinfections in lungs (**B**) and livers (**C**) of wildtype and S100A9^−/−^ mice harvested at 36 hpi with either wildtype (black symbols) or Δ*znuB* (open symbols) *A. baumannii.* Each symbol represents one animal. The data were combined from two independent experiments with 5–10 mice per experiment per genotype.
(TIF)
