# Correction: Identification of an *Acinetobacter baumannii* Zinc Acquisition System that Facilitates Resistance to Calprotectin-mediated Zinc Sequestration

**DOI:** 10.1371/annotation/2968451e-04b8-4705-bee9-9e40bceffe67

**Published:** 2013-01-11

**Authors:** M. Indriati Hood, Brittany L. Mortensen, Jessica L. Moore, Yaofang Zhang, Thomas E. Kehl-Fie, Norie Sugitani, Walter J. Chazin, Richard M. Caprioli, Eric P. Skaar

The following information was missing from the Funding section: B.L.M. was supported by the Childhood Infections Research Program (ChIRP) training program (1T32AI095202-01).

