# Supplementary figures and images for: Identification of an Acinetobacter baumannii Zinc Acquisition System that Facilitates Resistance to Calprotectin-mediated Zinc Sequestration
Source: PLoS Pathog. 2012 Dec 6;8(12):e1003068. doi: 10.1371/journal.ppat.1003068 (PMC3516566; doi:10.1371/journal.ppat.1003068)

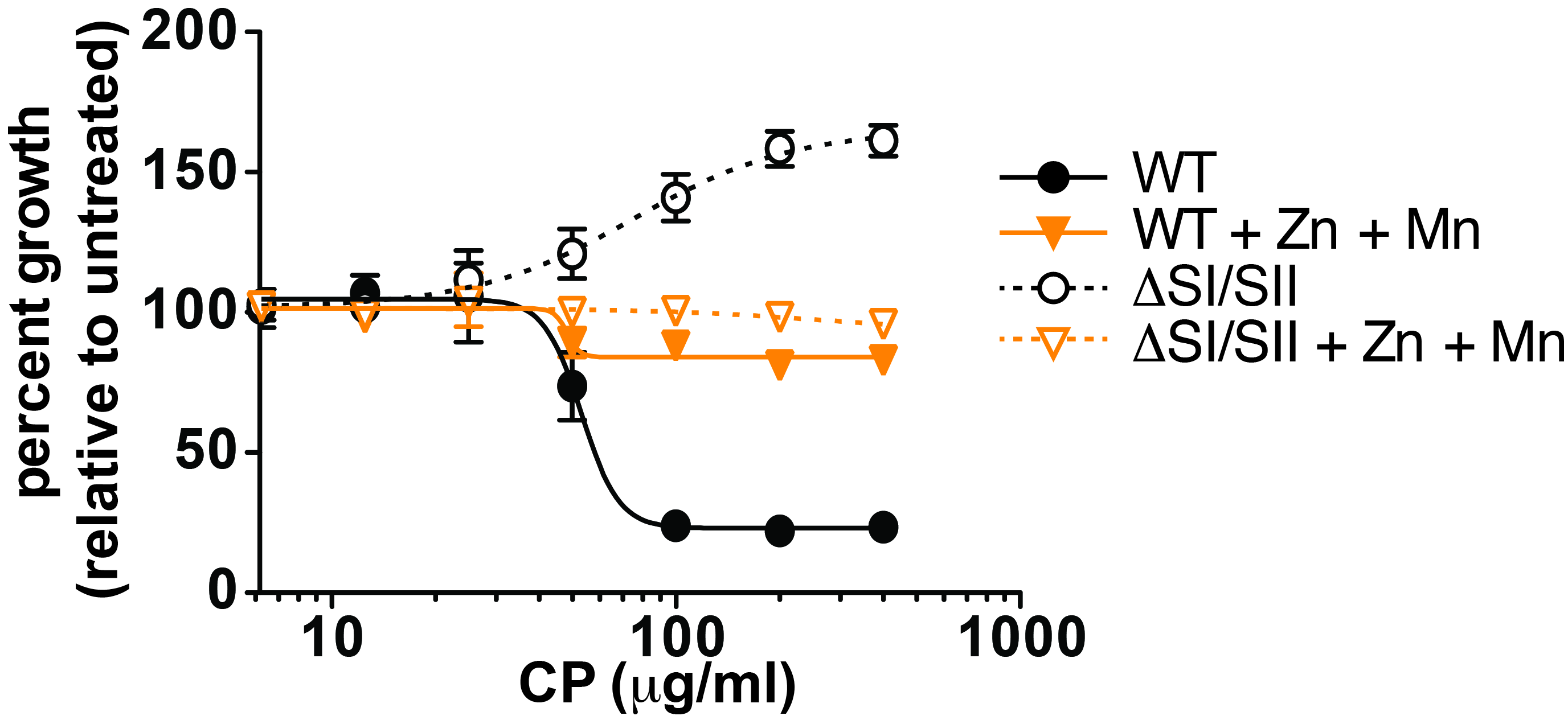

Supplement: Figure S1 — A. baumannii growth in the presence of increasing concentrations of CP (solid lines) or ΔSI/SII, a variant of CP that no longer binds Mn and Zn (dashed lines). Black indicates no Mn or Zn added, while orange indicates 25 µM Mn and 25 µM Zn added back. Data represent the average of three biological replicates. (TIF) [file ppat.1003068.s001.tif]

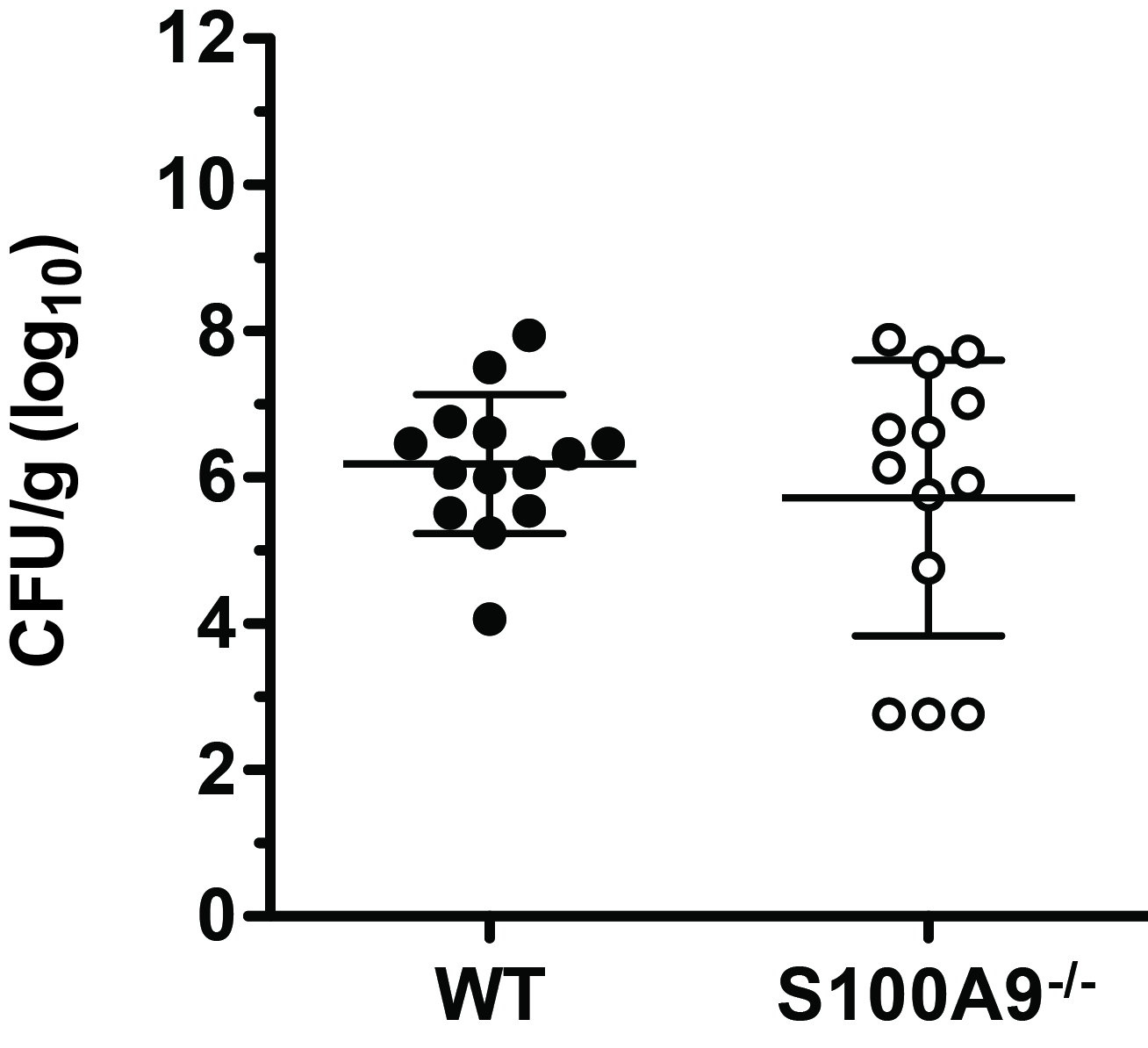

Supplement: Figure S2 — Bacterial burdens in lungs of wildtype and S100A9−/− mice harvested at 72 hpi with A. baumannii. Each symbol represents one animal. Only data from mice surviving to 72 hours are shown. The data were combined from two independent experiments with at least 10 mice per experiment per genotype. (TIF) [file ppat.1003068.s002.tif]

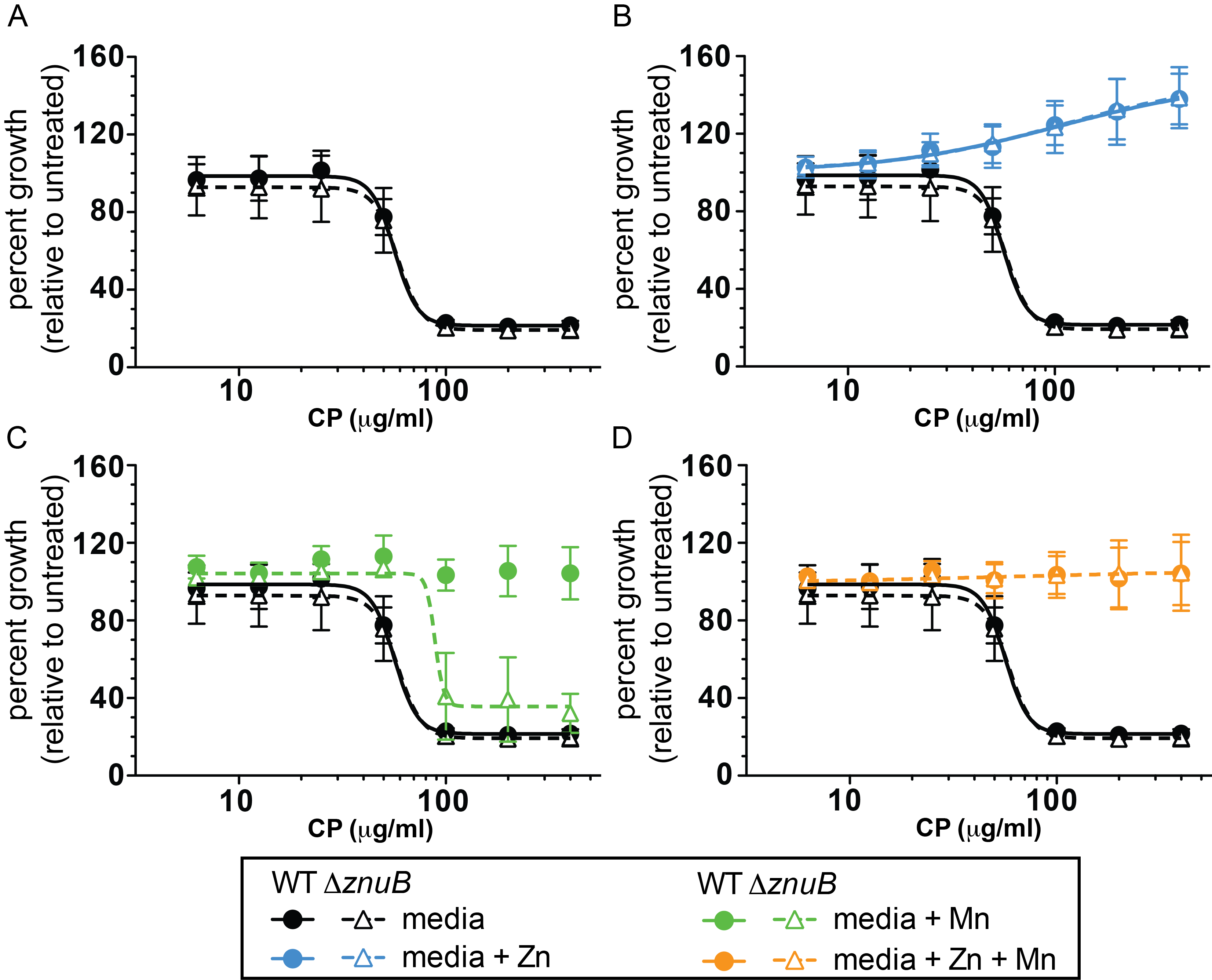

Supplement: Figure S3 — CP growth inhibition assays comparing wildtype with ΔznuB. Bacteria were cultured in CP growth media in the presence of increasing concentrations of CP without supplementation (A) or with addition of 25 µM Zn (B), 25 µM Mn (C) or both 25 µM Zn and 25 µM Mn (D). Data are presented as the percent growth relative to bacterial growth without CP. Curve fit was performed using a non-linear regression with variable slope. Curves are not drawn for WT+Mn and WT+Zn and Mn because these data are not converged and therefore the same curve fit parameters could not be used. Data are the average of three independent experiments with at least three biological replicates each. (TIF) [file ppat.1003068.s003.tif]

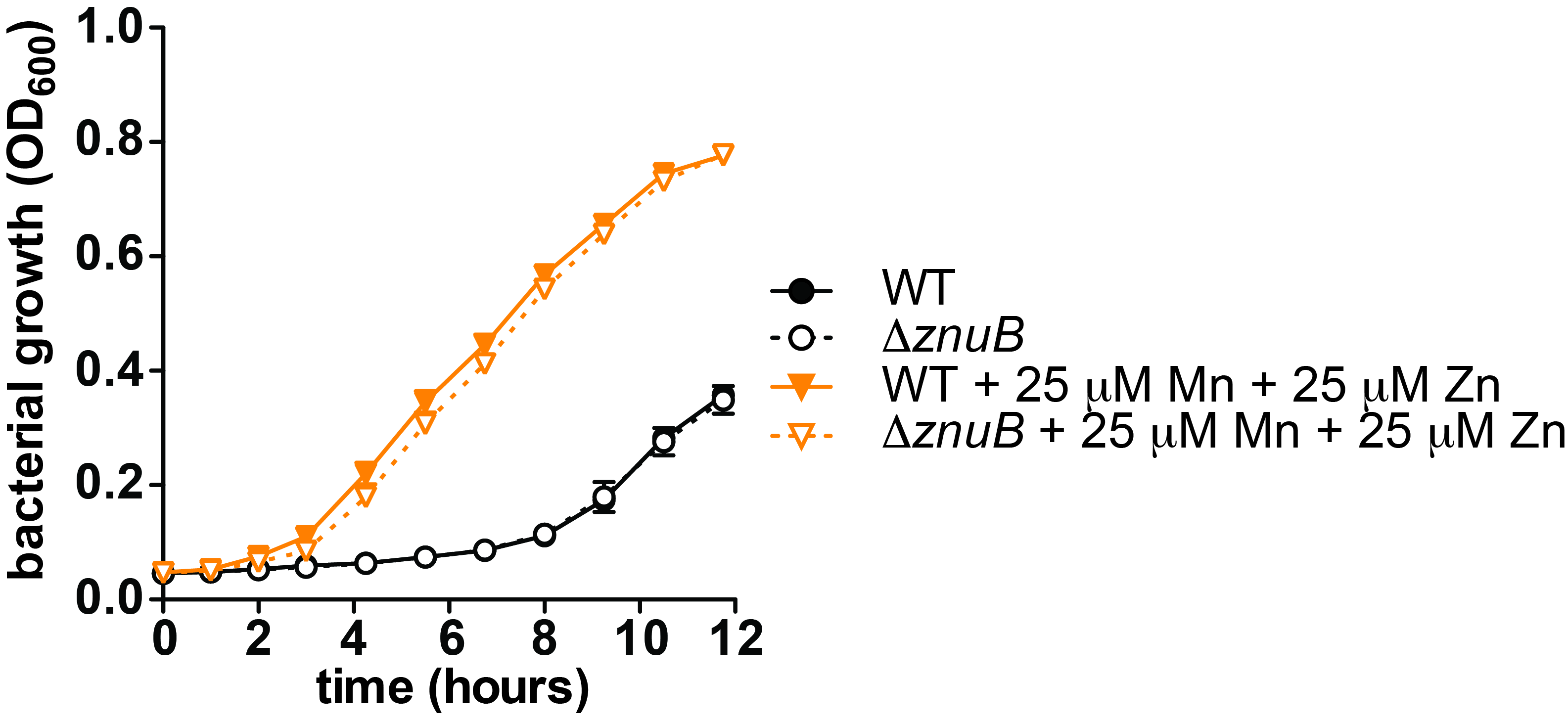

Supplement: Figure S4 — Growth curve analyses of wildtype and ΔznuB cultured in CP growth media with (orange) or without (black) added Mn and Zn. Data are averaged from at least three biological replicates. Error bars, which may be obscured by the symbols in some cases, represent one standard deviation from the mean. (TIF) [file ppat.1003068.s004.tif]

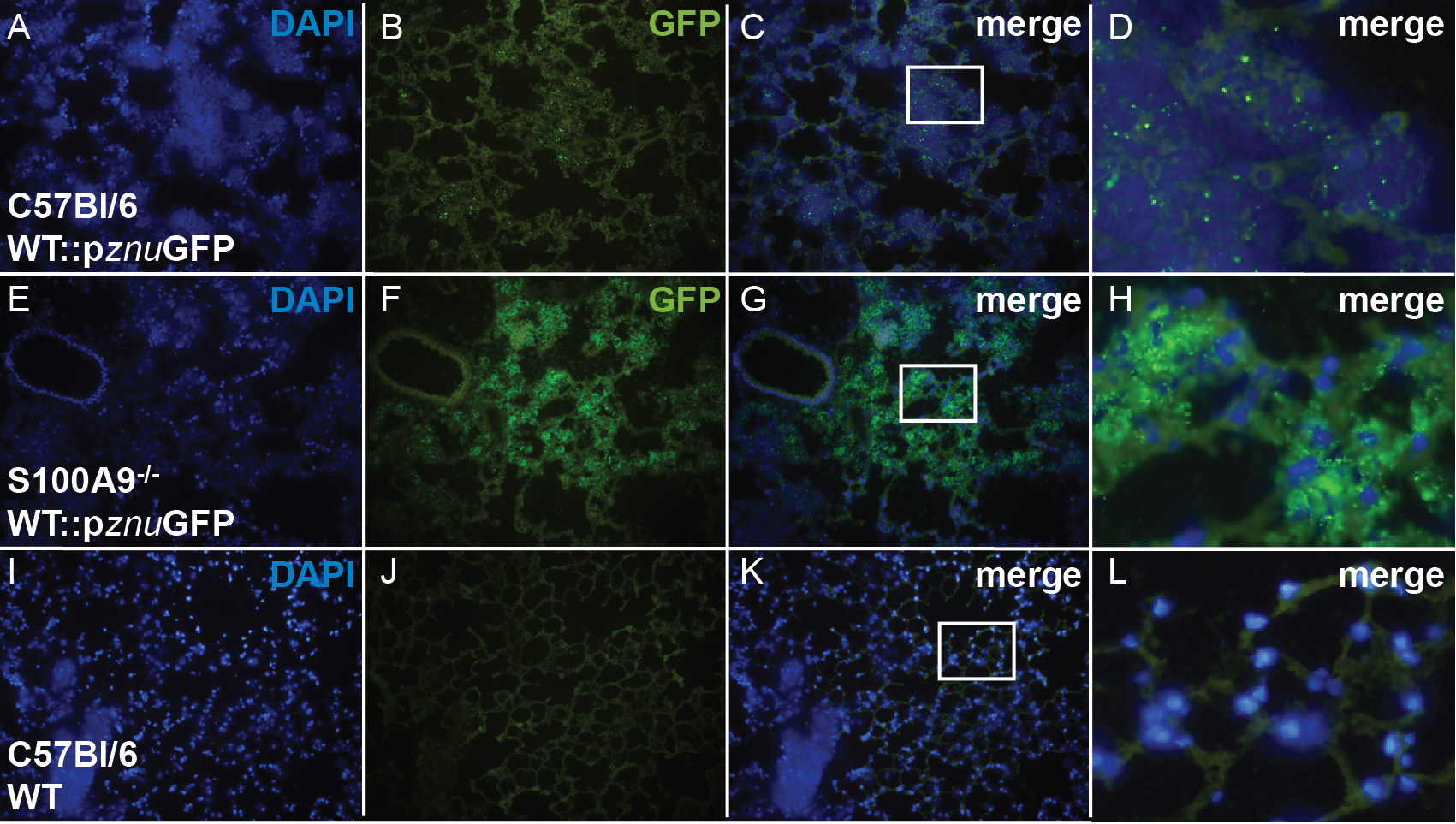

Supplement: Figure S5 — In vivo GFP expression driven by the znu promoter. Frozen sections of lungs harvested at 36 hpi from wildtype (A–D) or S100A9−/− (E–H) mice infected with WT::pznuGFP and stained with DAPI. Sections from lungs of mice infected with wildtype bacteria without the pznuGFP plasmid (I–L) are shown for comparison. (TIF) [file ppat.1003068.s005.tif]

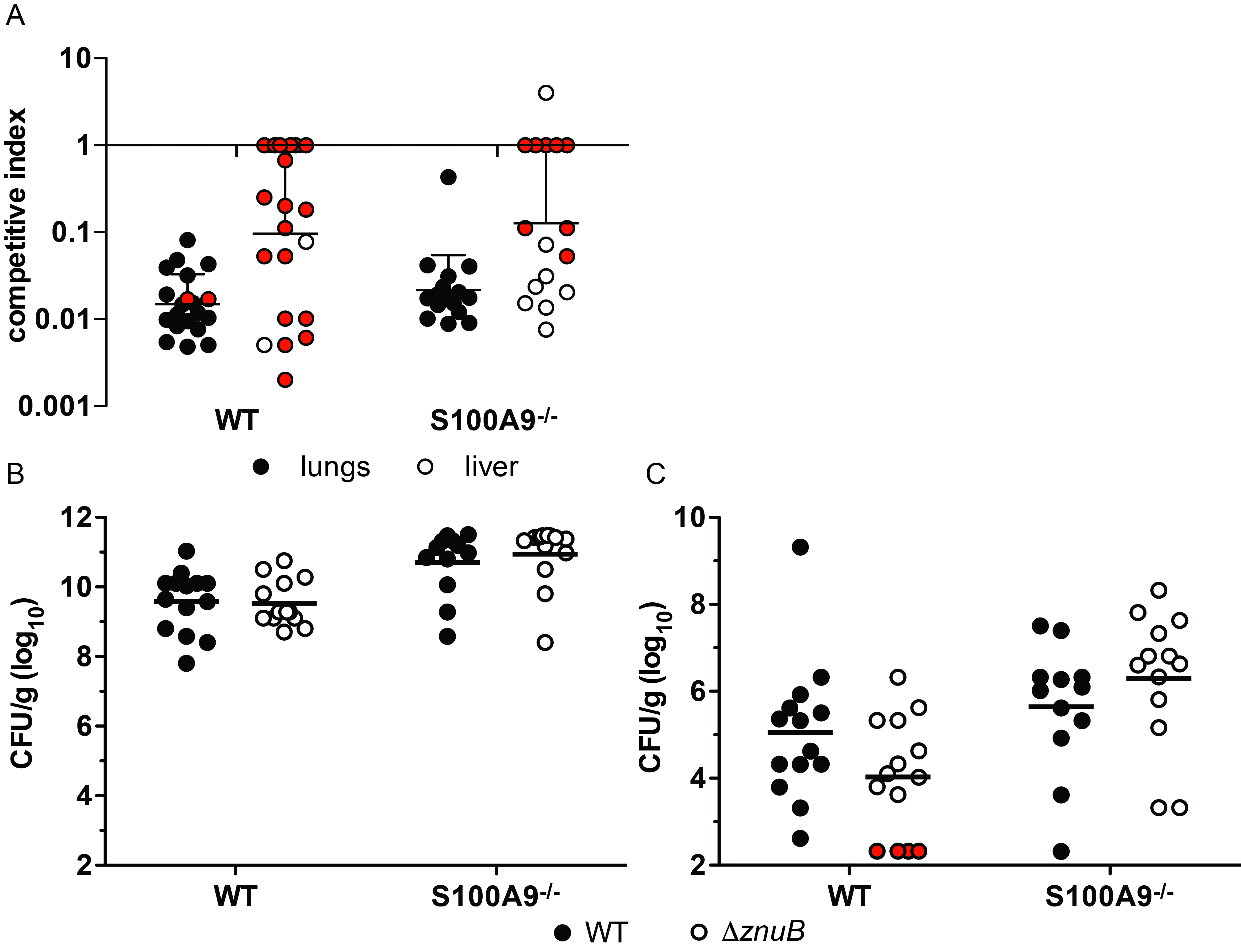

Supplement: Figure S6 — (A) Competitive indices of ΔznuB compared to wildtype A. baumannii in lungs and livers of wildtype and S100A9−/− mice. Each symbol represents one animal. Red symbols indicate mice in which the competitive index was determined by setting the recovered CFU for ΔznuB at the limit of detection since there were no recoverable CFU of ΔznuB in these mice. (B–C) Bacterial burdens from monoinfections in lungs (B) and livers (C) of wildtype and S100A9−/− mice harvested at 36 hpi with either wildtype (black symbols) or ΔznuB (open symbols) A. baumannii. Each symbol represents one animal. The data were combined from two independent experiments with 5–10 mice per experiment per genotype. (TIF) [file ppat.1003068.s006.tif]
